# AST/ALT-to-platelet ratio (AARPRI) predicts gynaecological cancers: a 8-years follow-up study in 653 women

**DOI:** 10.1038/s41598-023-44243-y

**Published:** 2023-10-18

**Authors:** Lucilla Crudele, Carlo De Matteis, Giusi Graziano, Fabio Novielli, Stefano Petruzzelli, Elena Piccinin, Raffaella Maria Gadaleta, Marica Cariello, Antonio Moschetta

**Affiliations:** 1 Department of Interdisciplinary Medicine, University of Bari “Aldo Moro”, Piazza Giulio Cesare n. 11, 70124 Bari, Italy; 2 Center for Outcomes Research and Clinical Epidemiology (CORESEARCH), 65124 Pescara, Italy; 3 Department of Translational Biomedicine and Neuroscience (DiBraiN), University of Bari “Aldo Moro”, Bari, Italy; 4 INBB, National Institute for Biostructures and Biosystems, 00136 Rome, Italy

**Keywords:** Cancer, Predictive markers, Liver fibrosis, Non-alcoholic fatty liver disease

## Abstract

Non-alcoholic fatty liver disease (NAFLD), specifically liver steatosis and fibrosis with steatohepatitis (NASH), is often associated with visceral adiposopathy, whose pathogenetic features have been proposed as tumorigenic triggers. We performed a prospective analysis in 653 metabolic women to reveal any conditions that may predict and concur to cancer development during a 8-years period of follow-up. Among clinical and biochemical variables, only AST and non-invasive liver fibrosis scores (AARPRI, APRI, FIB-4, mFIB4) significantly distinguished cancer-developer women (n = 62, 9.5%) from those who did not develop cancer (p < 0.001). In ROC analysis, these scores also showed good sensitivity and specificity in differentiating women who developed cancer (all p < 0.001). We then calculated OR for these indexes finding that increased AARPRI was associated with the highest risk (OR = 6, p < 0.001) of gynaecological cancers development. We further validated these cut-off values in women who had developed other types of cancer, confirming that AARPRI is able to identify the risk for cancer development (OR = 5, p < 0.001). Our findings support the hypothesis that NAFLD, more than obesity per se, is directly associated with the clinical and pathogenic metabolic scenario of gynaecological cancers and encourage the use of liver fibrosis indexes to detect risk of cancer onset in women. Preventing adiposopathy and NAFLD through lifestyle and therapies may represent an instrumental strategy for cancer prevention and/or co-treatment in oncology.

## Introduction

Cancer is a worldwide challenge from a clinical perspective as well as for policy makers and epidemiologists. Indeed, the increasing spread of risk factors, such as unhealthy lifestyles and dieting, together with novel powerful diagnostic instruments, led to record an increased cancer incidence^1^.

Visceral obesity, and more specifically adiposopathy, is involved in tumorigenesis and strictly linked to worse cancer prognosis by causing low grade-chronic inflammation, insulin resistance, and fatty liver^2^. Intriguingly, it is particularly true for those types of cancer whose incidence and resistance to treatment increase when dysmetabolic conditions come into play^3^. In women, breast, ovarian and uterus cancers are among the most common obesity-related tumors and are burdened by high mortality and morbidity rates^3^. By focusing on sex-specific metabolic and hormonal features, gender medicine is now aiming to reach a more personalized clinical approach between males and females. This will help to explain why males and females exposed to identical risk factors may display a different cancer incidence, as well as the different response and sensitivity to the same treatment.

While blood work and non-invasive tests are easy to use to evaluate systemic inflammation and insulin resistance, the assessment of the severity of hepatocytes fat accumulation, typical of non-alcoholic fatty liver disease (NAFLD), as well as steatosis and fibrosis characterizing steatohepatitis (NASH), is based on an “imperfect” gold standard test represented by liver biopsy an invasive procedure, difficult to perform in a routinary clinical setting. For these reasons, some non-invasive and easy-to-use scores have been recommended by clinical guidelines in individuals with fatty liver and suspected advanced fibrosis to perform appropriate risk stratification and predict morbidity and mortality. In recent years, these non-invasive liver fibrosis scores have been also proposed for the prediction of a wide range of conditions, including cardiovascular risk^4^, SARS-CoV-2 infection severity^5^, and hepatic and extra-hepatic cancers^6^. Moreover, although large validation studies are still lacking, these scores also seem to have an additional value in predicting the development of hepatic and extra-hepatic cancers^7^.

In this observational prospective study, we followed-up a cohort of 653 metabolic women without previous oncological history for 8 years to find any baseline specific conditions of risk or prediction that could drive their susceptibility for cancer development. Thus, in addiction to anthropometric assessment and bio-humoral parameters, we also calculated non-invasive scores such as APRI (AST to Platelet Ratio Index), FIB-4 (Fibrosis-4 index), modified FIB-4 (mFIB-4), and AARPRI [(AST to ALT ratio) to Platelet Ratio Index] (see “Methods”).

## Results

### Cancer onset during the observational period

**Figure 1:**
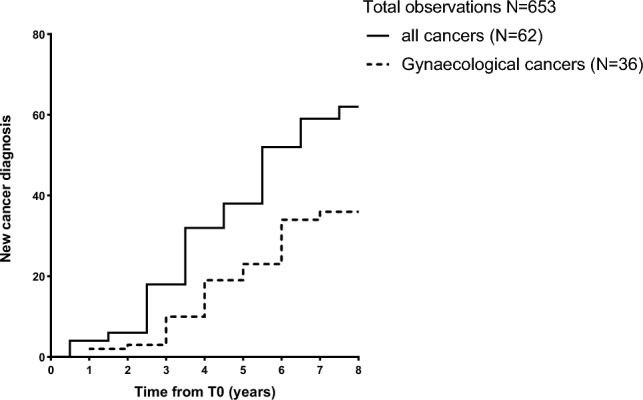
New cancer diagnosis during the follow-up period. New cancer cases have been recorded during a follow-up period up to 8 years. Patients first assessment was performed from January 2014 to December 2017. Follow-up was performed every year, by phone or clinical evaluation according to clinical conditions and Institutional policy for patients’ prevalent disease, and concluded in December 2022.

During the follow up, 62 patients (9.5%) developed cancer. Among them, 36 women developed gynaecological cancers (uterine, breast, and ovarian). The most frequent types of cancer were uterus (n = 16), breast (n = 11), thyroid (n = 10), and ovarian (n = 9). 6 patients developed colorectal cancer and 4 patients developed stomach cancer, 2 patients were diagnosed with melanoma, and single cases of hepatocellular carcinoma, pancreas cancer and Acute Myeloid Leukaemia were recorded. One patient only referred cancer onset without specifying the type. None of them had cancer-associated known genetic mutations. Figure 1 shows the timeline of new-cancer onset during the follow-up period.

### Clinical characterization of the study population

Baseline characteristics of 653 female patients are shown in Table 1. Mean age of our cohort was 55.2 ± 0.6 years. Intriguingly, most of our patients were dysmetabolic, since 513 (79%) showed a Waist Circumference (WC) value above the cut-off of 80 cm (diagnostic criterion for Metabolic Syndrome) and mean WC value was 94.7 ± 0.6 cm. This characterization was confirmed also when considering Body Mass Index (BMI) (mean value 26.8 ± 0.3), since 334 (51%) patients were overweight and 170 (26%) presented with obesity condition.

**Table 1:** Metabolic characterization of the study population and cancer detection during the follow-up.

Study population	N = 653
Age (years)	55.2 ± 0.6
Waist Circumference (cm)	94.7 ± 0.6
BMI (Kg/sqm)	26.8 ± 0.3
FPG (mg/dL)	97.0 ± 1.2
HbA1c (mmol/mol)	40.9 ± 0.6
AST (U/L)	22.8 ± 0.4
ALT (U/L)	28.6 ± 0.6
GGT (U/L)	29.1 ± 1.2
ALP (U/L)	70.8 ± 1.0
Total cholesterol (mg/dL)	190.0 ± 1.7
HDL cholesterol (mg/dL)	59.1 ± 0.6
LDL cholesterol (mg/dL)	108.6 ± 1.4
Triglycerides (mg/dL)	109.1 ± 2.5
WC positive criterion	513 (79%)
BMI ≥ 25 kg/sqm	334 (51%)
BMI ≥ 30 kg/sqm	170 (26%)
No cancer developers	591 (90%)
Cancer developers	62 (10%)
Uterus	16
Breast	11
Thyroid	10
Ovarian	9
Colorectum	6
Stomach	4
Melanoma	2
Liver	1
Pancreas	1
Acute Myeloid Leukaemia	1
Not specified	1

Positive criterion for WC (Waist Circumference) was ≥ 80 cm in females according to IDF (international Diabetes Federation) Metabolic Syndrome diagnosis. Data is expressed as mean ± SD (Standard Deviation) for numerical data, in counts and percentages for categorical data.

BMI, body mass index; FPG, fasting glucose plasma; HbA1c, glycosylated haemoglobin; AST, aspartate transferase; ALT, alanine transferase; GGT, gamma-glutamyl transpeptidase; ALP, alkaline phosphatase.

### Clinical characterization of cancer population and comparisons with no cancer group

When comparing bio-humoral and clinical parameters between patients who developed cancer and women who did not (Table 2), no significant differences were found at baseline, except for AST levels (36.8 ± 2.5 U/I *vs* 21.9 ± 8.2 U/I, p-value (p) < 0.001).

**Table 2:** Comparison of clinical and biochemical variables between patients who developed and did not develop cancer during the follow-up period.

	No cancer (n = 591)	Cancer (n = 62)	*p*-value
Age (years)	55.1 ± 15.8	54.9 ± 12.8	ns
BMI (Kg/sqm)	26.7 ± 6.4	26.4 ± 5.5	ns
Waist Circumference (cm)	94.7 ± 15.2	95.0 ± 13.4	ns
FPG (mg/dL)	96.4 ± 28.8	104.1 ± 47.5	ns
AST (U/L)	21.9 ± 8.2	36.8 ± 2.5	< 0.001
ALT (U/L)	28.6 ± 16.0	29.0 ± 12.6	ns
GGT (U/L)	29.1 ± 30.0	28.4 ± 25.1	ns
Total Cholesterol (mg/dL)	189.5 ± 41.5	194.2 ± 39.2	ns
HDL Cholesterol (mg/dL)	59.1 ± 15.1	60.0 ± 17.7	ns
LDL Cholesterol (mg/dL)	108.2 ± 33.5	110.7 ± 34.1	ns
Triglycerides (mg/dL)	108.5 ± 63.9	113.0 ± 53.0	ns
Platelet Count (× 10^3^/L)	251.7 ± 61.5	241.7 ± 85.7	ns
AARPRI score	0.5 ± 0.3	0.9 ± 0.6	< 0.001
APRI score	0.3 ± 0.1	0.5 ± 0.3	< 0.001
FIB-4 score	1.0 ± 0.6	1.8 ± 1.3	< 0.001
mFIB-4 score	2.0 ± 1.3	3.4 ± 2.6	< 0.001

Data is presented as mean ± SD (Standard Deviation). Comparisons were performed by Mann–Whitney U Test, and statistical significance was assessed for *p*-values < 0.05.

BMI, body mass index; FPG, fasting glucose plasma; AST, aspartate transferase; ALT, alanine transferase; GGT: gamma-glutamyl transpeptidase; ALP, alkaline phosphatase; AARPRI, (AST to ALT ratio) to platelet ratio index; APRI, AST-platelet ratio index; mAPRI, modified APRI; FIB-4, fibrosis-4 index; mFIB-4, modified FIB-4.

**Figure 2:**
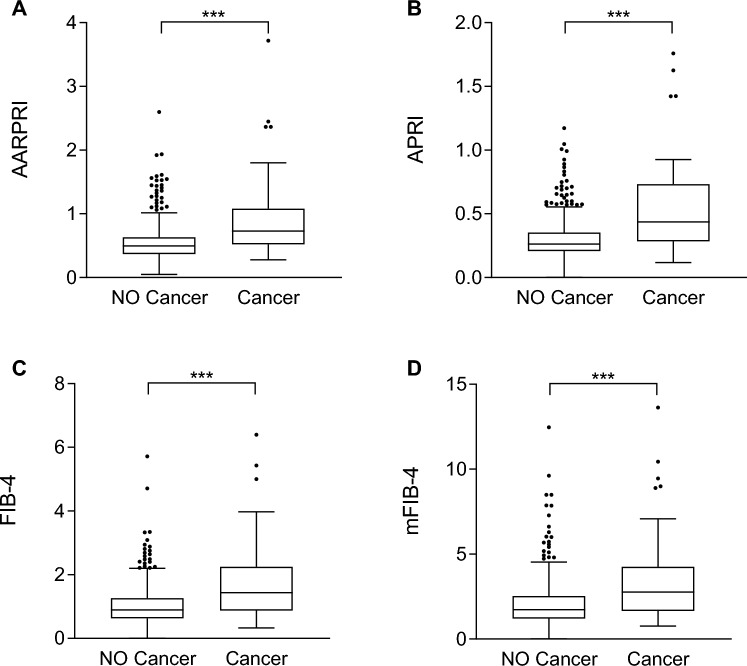
Non-invasive hepatic fibrosis scores comparisons between women who developed cancer and women who did not during the follow-up period. Comparisons between AARPRI (**A**), APRI (**B**), FIB-4 (**C**), mFIB-4 (**D**) values in patients who developed (n = 62) and did not develop (n = 591) cancer during the follow-up period. Statistical significance was assessed by Mann–Whitney U Test, p < 0.05 were considered significant (***p < 0.001). The box plots show the median (second quartile), first and third quartile, Tukey whiskers represents 1.5 times the interquartile distance or to the highest or lowest point, whichever is shorter. Any data beyond these whiskers are shown as points. AST, aspartate transaminases; AARPRI, (AST to ALT ratio) to Platelet Ratio Index; APRI, AST-platelet ratio index; FIB-4, fibrosis-4 index; mFIB-4, modified FIB-4.

Thus, we compared AARPRI, APRI, FIB-4, mFIB-4 between developer and not-developer cancer women. We found that AARPRI was significantly increased (0.5 ± 0.3 *vs* 0.9 ± 0.6, p < 0.001) in patients who developed cancer during the follow-up period (Fig. 2A), as well as APRI (0.3 ± 0.1 *vs* 0.5 ± 0.3, p < 0.001, Fig. 2B), FIB-4 (1.0 ± 0.6 *vs* 1.8 ± 1.3, p < 0.001, Fig. 2C) and mFIB-4 (2.0 ± 1.3 *vs* 3.4 ± 2.6, p < 0.001, Fig. 2D). These data underscore the *bona fide* predictive power of these scores as well as the impact of NAFLD- associated dysmetabolic conditions in cancers development.

### Non-invasive liver fibrosis scores distinguished women who developed specific types of cancer

Since some non-invasive scores of liver fibrosis have already showed a stronger association with specific types of cancer^8^, and given the fact that in our cohort these scores distinguished cancer developers, we then performed multiple comparisons among women who developed the most represented types of cancer (uterus, breast, thyroid, and ovarian). Intriguingly, we observed that, differently from other clinical and bio-humoral parameters, only AARPRI, APRI, FIB-4, and mFIB-4 were significantly different (p < 0.001) among these subgroups (Table 3). Furthermore, also when comparing single cancer subgroups with women who did not develop cancer, we found that all these scores were significantly higher in cancer patients, thus further supporting the use of these indexes in the prediction of cancer and the involvement of NAFLD in its pathogenesis.

**Table 3:** Comparison of clinical and biochemical variables between patients who did not develop cancer and those who developed specific types of cancer during the follow-up period.

	No cancer (n = 591)	Uterus cancer (n = 16)	Breast cancer (n = 11)	Thyroid cancer (n = 10)	Ovarian cancer (n = 9)	ANOVA p-value
Age (years)	55.1 ± 15.8	57.0 ± 1.0	53.3 ± 9.7	55.9 ± 14.9	58.8 ± 8.8	ns
BMI (Kg/sqm)	26.7 ± 6.4	28.3 ± 5.1	26.0 ± 5.4	27.2 ± 5.5	26.1 ± 6.4	ns
Waist Circumference (cm)	94.7 ± 15.2	93.2 ± 14.2	96.1 ± 14.4	94.3 ± 11.6	94.5 ± 13.5	ns
FPG (mg/dL)	96.4 ± 28.8	100.1 ± 22.4	99.4 ± 35.7	99.2 ± 36.4	126.1 ± 80.5*	0.06
Total Cholesterol (mg/dL)	189.5 ± 41.5	196.1 ± 45.9	199.0 ± 43.8	192.5 ± 47.3	201.2 ± 34.5	ns
HDL Cholesterol (mg/dL)	59.1 ± 15.1	55.9 ± 11.0	59.4 ± 27.3	62.6 ± 16.2	65.6 ± 11.1	ns
LDL Cholesterol (mg/dL)	108.2 ± 33.5	117.6 ± 41.9	109.9 ± 42.0	109.0 ± 36.0	113.8 ± 35.0	ns
Triglycerides (mg/dL)	108.5 ± 63.9	108.5 ± 42.9	138.4 ± 62.6	105.3 ± 64.7	108.9 ± 46.8	ns
AARPRI score	0.5 ± 0.3	0.7 ± 0.4*	1.2 ± 1.0*	0.9 ± 0.5*	1.3 ± 0.8*	< 0.001
APRI score	0.3 ± 0.1	0.4 ± 0.2*	0.6 ± 0.5*	0.5 ± 0.2*	0.7 ± 0.4*	< 0.001
FIB-4 score	1.0 ± 0.6	1.5 ± 0.8*	2.1 ± 2.0*	1.5 ± 0.9*	2.4 ± 1.5*	< 0.001
mFIB-4 score	2.0 ± 1.3	2.8 ± 1.6	4.3 ± 4.1*	3.0 ± 1.9*	4.9 ± 3.1*	< 0.001

Data is reported as mean ± SD (Standard Deviation). Comparisons between No cancer group and specific type-cancer groups were performed by Kruskal–Wallis One-Way ANOVA and p-values < 0.05 were considered significant. (*) represents significant difference with NO cancer group after Fisher’s LSD Multiple-Comparison Test.

BMI, body mass index; FPG, fasting glucose plasma; AARPRI,(AST to ALT ratio) to platelet ratio index; APRI, AST-platelet ratio index; FIB-4, fibrosis-4 index; mFIB-4, modified FIB-4.

### Non-invasive scores in prediction of gynaecological cancers

In view of an interesting theory pertaining to complex effects of sex and sex hormones on human NAFLD^9^, we then considered only gynaecological cancers (uterus, breast and ovarian) to investigate if their risk is increased in fatty liver condition. Thus, we performed receiver operating characteristic (ROC) curves and cut-off analysis of non-invasive liver fibrosis indexes for prediction of sex specific female cancers.

**Figure 3:**
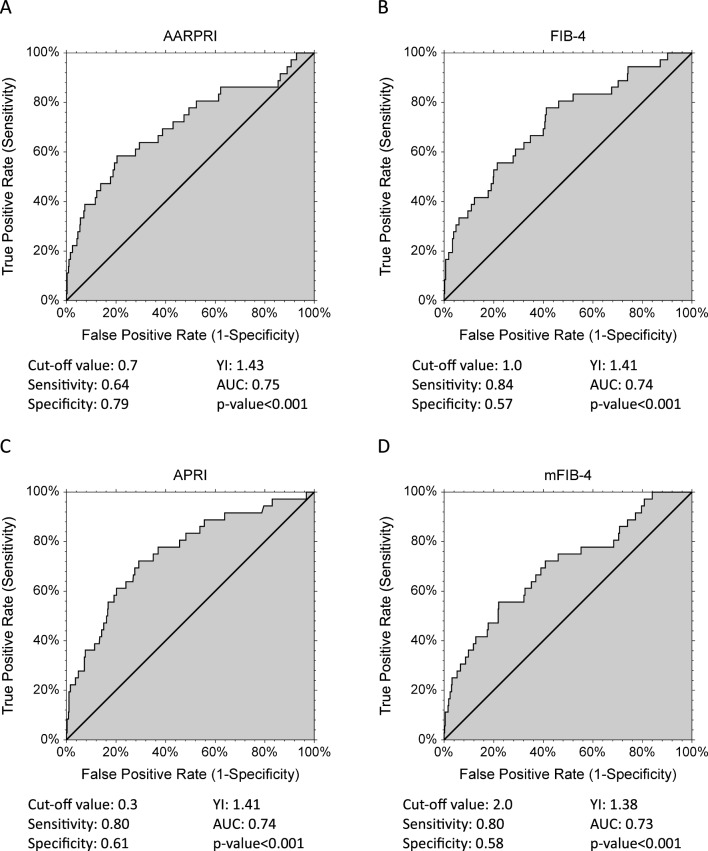
ROC reports for gynaecological (uterus, breast, ovarian) cancer discrimination. Cut-off values with related sensitivity and specificity levels and empirical estimation of area under curve (AUC) are shown under each graph for AARPRI (**A**), FIB-4 (**B**), APRI (**C**), and mFIB-4 (**D**). YI, Youden’s Index; AARPRI, (AST to ALT ratio) to Platelet Ratio Index; FIB-4, fibrosis-4 index; APRI, AST-platelet ratio index; mFIB-4, modified FIB-4.

As shown in Fig. 3A, to all, AARPRI = 0.7 showed the best Youden’s Index (sensitivity 0.64 and specificity 0.79) and the larger Area under the Curve (AUC) (0.75, p < 0.001). FIB-4 (Fig. 3B) also showed a good Youden’s Index (calculated cut-off of 1.1, sensitivity 0.84 and specificity 0.57) and a satisfying AUC (0.74, p < 0.001). Similarly, although with lower AUC and Youden’s Index, APRI (Fig. 3C) and mFIB-4 (Fig. 3D) ROCs predicted female cancers.

### Non-invasive liver fibrosis scores as risk predictors of gynaecological cancer

**Figure 4:**
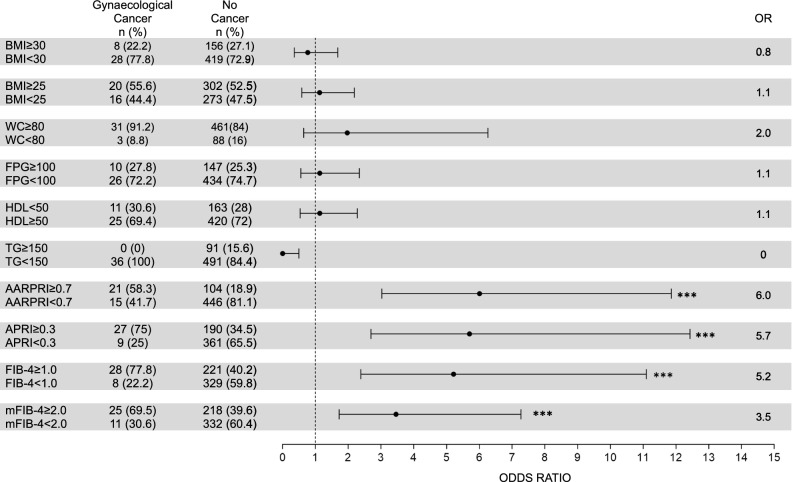
Risk for gynaecological (uterus, breast, ovarian) cancers development. For WC, FPG, HDL, and TG we considered cut-off values used for Metabolic Syndrome IDF diagnosis. For AARPRI, APRI, FIB-4, and mFIB-4 we considered cut-off values computed in ROC analysis (see Fig. 3). Each row shows the contingency table to assess the Odds Ratio (OR) and detect the association of single dysmetabolic conditions with gynaecological cancers development. For each variable, OR representation with 95% confidence interval is provided. (***) represents p < 0.001. BMI, body mass index; WC, waist circumference; FPG, fasting plasma glucose; TG, triglycerides; AARPRI, (AST to ALT ratio) to platelet ratio index; APRI, AST-platelet ratio index; FIB-4, fibrosis-4 index; mFIB-4, modified FIB-4.

Since all considered scores ultimately differentiated women who developed female specific cancers from those who did not develop any kind of cancer, we calculated the associated Odd Ratio (OR) for gynecological cancer, using our ROC-calculated cut-offs as discriminants (Fig. 4).

Among patients who developed female cancers, 21 (58.3%) showed a AARPRI ≥ 0.7, while 446 (81.1%) of no cancer population had AARPRI < 0.7. Thus, the AARPRI associated OR was 6 (p < 0.001). Among female cancers developers, 27 (75%) showed a APRI ≥ 0.3, while 361 (65.5%) of no cancer population had APRI < 0.3 and the APRI associated OR was 5.7 (p < 0.001). FIB-4 showed an associated OR of 5.2 (p < 0.001) since 28 (77.8%) of cancer patients and 221 (40.2%) of no-cancer ones had FIB-4 ≥ 1. Regarding mFIB-4, the associated OR was 3.5 (p < 0.001).

To compare the association of non-invasive liver fibrosis scores and other routinary metabolic parameters with female cancer development, we also calculated OR for increased BMI (overweight and obesity conditions), WC, fasting plasma glucose (FPG), and triglycerides (TG) as well as for reduced HDL-cholesterol levels, using the IDF MetS cut-off criteria. Any of them exhibited a significant association with cancer development, since BMI ≥ 30 characterized only 8 (22.2%) women of cancer group (OR = 0.8, p = ns), BMI ≥ 25 was present in 20 (55.6%) female cancer patients and 302 (52.5%) non cancer women (OR = 1.1, p = ns). Increased WC showed a similar prevalence in both groups (91.2% in cancer developers and 84% in no cancer group) with an associated OR of 2 (p = ns). Regarding bio-humoral parameters, similar OR without significant p-values were detected (FPG OR = 1.1, HDL OR = 1.1, TG OR = 0).

### Validation of non-invasive liver fibrosis scores as risk predictors in not-gynaecological cancers

**Figure 5:**
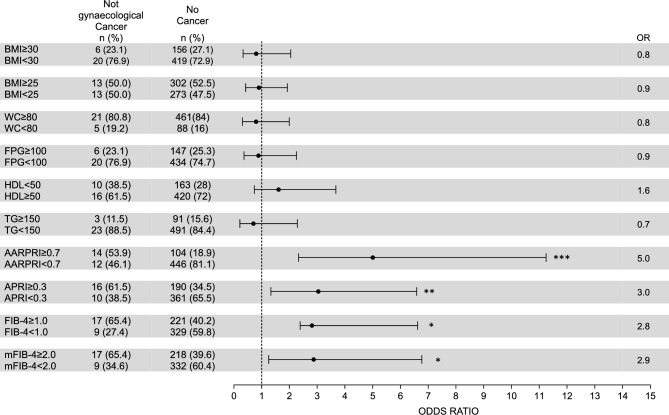
Risk for not gynaecological cancers development. For WC, FPG, HDL, and TG we considered cut-off values used for IDF Metabolic Syndrome diagnosis. For AARPRI, APRI, FIB-4, and mFIB-4 we considered cut-off values computed in ROC analysis (see Fig. 3). Each row shows the contingency table to assess the Odds Ratio (OR) and detect the association of single dysmetabolic conditions with not gynaecological cancer development. For each variable, OR representation with 95% confidence interval is provided. (*) represents p < 0.05, (**) p < 0.01, (***) p-value < 0.001. BMI, body mass index; WC, waist circumference; FPG, fasting plasma glucose; TG, triglycerides; AARPRI, (AST to ALT ratio) to platelet ratio index; APRI, AST-platelet ratio index; FIB-4, fibrosis-4 index; mFIB-4, modified FIB-4.

Since for liver fibrosis indexes we used cut-off values based on our population of women who developed only gender specific cancers, to ultimately validate our cut-offs of liver fibrosis scores, we assessed ORs and analyzed the strength of their association with other types of cancers (Fig. 5).

Once again, differently from increased BMI (OR = 0.8 BMI ≥ 30 and 0.9 for BMI ≥ 25, p = ns), WC (OR = 0.8, p = ns), FPG (OR = 0.9, p = ns), TG (OR = 0.7, p = ns), and decreased HDL (OR = 1.6, p = ns), non-invasive liver fibrosis scores showed a significant association with cancer development. APRI ≥ 0.3 presented significant association with increased oncological risk (OR = 3, p < 0.01), as well as FIB-4 (OR = 2.8) and mFIB-4 (OR = 2.9) with p < 0.05. AARPRI showed the best OR value (5.00) and the most significant association (p < 0.001), further confirming our study.

## Discussion

In this study, we describe for the first time that liver fibrosis scores are able to predict gynaecological cancers (breast, uterus and ovarian) in metabolic women and could be also used to assess the risk of other cancers development.

In patients with NAFLD, which could be associated to MetS, systemic and abdominal obesity, the risk of liver-related mortality increases with the grade of liver fibrosis and NASH^10^. Visceral adiposopathy, the source of signaling factors and hormones prompting clinical complications of obesity, has also been correlated with cancer development^11^ and worse prognosis^2^ from both an epidemiological and pathogenic perspective^12^.

Increased FPG levels, together with a low-grade chronic inflammation which are both the main stigma of adiposopathy, seem to predispose the pro-tumoral macro- and micro-environment. Furthermore, a derangement in cholesterol metabolism with increased intra-tumoral cholesterol levels, has also been proposed as fueling process for cancer cells growth^13^. Reduction of cholesterol excretion from cells with lower functional reverse cholesterol transport and lower HDL-cholesterol levels have been associated with endometrial as well as with breast cancer incidence^14^ and also used for hepatocellular cancer prediction^15^. Intriguingly, the cut-off of 40 mg/dL that we used to assess the reduced HDL levels-associated OR for cancer, was also found to discriminate women with better overall survival in breast cancer^16^ opening new perspective on HDL role, beyond the well-known involvement in preventing cardiovascular diseases^17^.

Moving on female-specific neoplasms, obesity is frequently associated with breast and ovarian cancers^18^. BMI shows a direct association with increased risk for ovarian, breast, and uterus cancer^2^ and 41% of uterine cancer has been attributed to excess weight^19^. Surprisingly, in our population the assessment of increased systemic and abdominal obesity per se, via BMI and WC values respectively, neither differentiates women who developed cancers from women who did not, nor significantly reflects the increased risk of gynecological cancers. Nevertheless, since subjects with a higher BMI and other concomitant metabolic risk factors present with an increased risk of NAFLD^20^, one could speculate that the increased risk for malignancy associated with obesity may be related to NAFLD^21^, as pointed by the high ORs for liver fibrosis scores shown in this study. Indeed, a recent meta-analysis also showed that NAFLD was associated with a nearly 40% higher risk of breast cancer and 60% higher risk for uterine and ovary^22^. Moving to other urogenital organs, also bladder cancer incidence is higher in obese patients with NAFLD^23,24^, and in obese women synchronous tumours of the urogenital tract are more common than one could expect^25^ leading to confusion during the diagnostic process.

NAFLD may be considered as a sexual dimorphic disease, with lower prevalence in women than in men and different metabolic features between sexes^26^. This epidemiological finding underlines the intimate relationship between estrogens and NAFLD and should prompt clinicians to apply different approaches in prevention and screening strategies in males and females. Indeed, among postmenopausal women with NAFLD, the duration of estrogen deficiency, associated with ovarian senescence, increases the risk of liver fibrosis^27^. This could be probably due to hypoestrogenism associated with hepatic steatosis through changes in the expression of genes involved in fat oxidation and lipogenesis^26^. While men and post-menopausal women are at risk of more severe hepatic fibrosis compared to pre-menopausal women, at any given degree of hepatocyte ballooning and portal inflammation estrogen replacement was associated with a 50% risk reduction of fibrosis^28^. On the other side, the same group found a distinct associations of gender, menopause, oral contraceptive use, and hormone replacement therapy with hepatic lobular inflammation, providing an interesting theory pertaining to complex effects of sex and sex hormones on human NAFLD, since despite increased liver injury and inflammation, premenopausal women showed a lower liver fibrosis compared with men and post-menopausal women^9^.

Female subjects with NAFLD had a significantly higher incidence rate of breast cancer^8^, and in a case–control study NAFLD was significantly associated with breast cancer independently from other traditional risk factors, even if this relationship was found only in the non-obese group^29^. A systematic review also found that the prevalence of NAFLD in adults with cancer appeared to be the highest in breast, gynecologic, and colorectal cancers^20^. Moreover, another meta-analysis found that for specific cancer site, the highest incidence rates in NAFLD patients were for uterine and breast cancer^30^. Similarly to our study design, a longitudinal cohort study evaluated 7413 patients recording 2224 incident cancers of which the most common type was breast, finding that women with NAFLD showed 2.2 incidence rate ratio for uterus cancer whilst ovarian cancer occurred more frequently in NAFLD at a young age^21^.

In the present study, during a follow-up period of 8 years, 9.5% of 653 women presented cancer. Non-invasive liver fibrosis scores (AARPRI, APRI, FIB-4, mFIB-4) significantly distinguished at index day cancer-developer women from those who did not develop cancer. Although we did not have data about elastography at time 0, non-invasive liver fibrosis indexes are biopsy-free scoring systems to assess the different stages of steatosis and fibrosis with the advantages of being accurate, non-invasive, and easily available^31^. Moreover, while liver steatosis diagnosed using ultrasound does not seem to be associated with cancer development, some non-invasive scores of liver fibrosis conversely showed a stronger association with it^8^. Among others, AARPRI values above the ROC cut-off of 0.7 were associated with a OR of 6 for gynaecological cancers development. The finding that, among all indexes, AARPRI shows the best strength in the assessment of cancer risk in women may depend on the variables that it considers. Differently from APRI, AARPRI considers AST to ALT ratio instead of AST, thus reducing the weight of AST elevation that occurs in severe hepatic inflammation. Furthermore, AARPRI does not consider age, differently from FIB-4 and mFIB-4, reducing the influence related to aging and associated chronic diseases. With regard to our AARPRI cut-off of 0.7, the same value has already shown to predict liver cirrhosis in chronic HBV patients^32^. Finally, to validate our cut-offs for non-invasive liver fibrosis indexes we also carried out ORs analysis in women who had developed non-gynecological cancers, confirming the significant utility of AARPRI and the others in prediction of cancer.

However, guidelines to standardize cut-off limits of these non-invasive scores are needed to improve test performance and clinical decision in NAFLD management^33,34^.

Our findings support the hypothesis that NAFLD, more than obesity per se, is directly associated with the clinical and pathogenic metabolic scenario of gynaecological cancers and encourage the use of liver fibrosis indexes to detect risk of cancer onset in women. The rapidly growing population of NAFLD women, should encourage counteracting adiposopathy through lifestyle and therapies as an instrumental strategy for gynaecological cancer prevention and/or co-treatment in oncology.

## Methods

### Aim, design and setting of the study

Patients’ recruitments, anthropometric, biochemical, and clinical variables were registered continuously in the electronic health register of Metabolic Diseases of the Department of Interdisciplinary Medicine—Internal Medicine Division—“Aldo Moro” University of Bari (Bari, Italy). A total of 656 female out-patients suspected of metabolic disorders (at least two positive criteria for MetS diagnosis or already diagnosed with T2D and/or NAFLD) were enrolled in this study at the beginning from January 2014 to December 2017. Patients with acute heart diseases (cardiac failure, coronary arterial disease, acute arrhythmias), renal and hepatic failure, infections, secondary hypertension, chronic systemic inflammatory diseases, and neoplastic diseases with recent onset (less than 10 years) and/or under chemotherapy were excluded.

Participants underwent physical examination, biochemical assessment, and abdomen ultrasound. To register the onset of new cancer cases, after this first assessment, follow-up was performed every year by phone or clinical re-evaluation, according to patients’ clinical conditions and Institutional policy for their prevalent disease and concluded in December 2022. Three patients were lost at follow-up, thus statistical analysis was performed on a final total population of 653 patients.

The study was approved by the Ethics Committee of the Azienda Ospedaliero-Universitaria Policlinico di Bari (Bari, Italy) in accordance with the requirements of the Declaration of Helsinki. Written informed consent for the use of clinical data was obtained from all participants in the study. In accordance with the approved Ethics Committee, only patients who were already 18 years old or more were included.

### Clinical assessment

All participants underwent a detailed anamnesis and physical examination. Anthropometric assessment was performed using standardized procedures. Briefly, WC was measured at the midpoint between the inferior part of the 12th costa and the anterior–superior iliac crest. BMI was computed as weight (Kg) divided by the height squared (sqm) and BMI values (Kg/sqm) 25.0–29.9 and over 30.0 were considered as overweight and obesity conditions, respectively. Ultrasound abdomen, when required, was performed with an Esaote My Lab 70 Gold ultrasound system with 2.5–5 MHz convex probes.

MetS was diagnosed according to International Diabetes Federation (IDF) definition^35^.

### Biochemical measurements

Morning blood samples were obtained after 12 h of fasting from the antecubital veins. After blood clotting and centrifugation, serum was processed for analysis of biochemical markers of glucose and lipid metabolism. Liver, renal, thyroid and inflammatory markers were studied following standardized biochemical procedures. All biochemical measurements were centralized and performed in the ISO 9001 certified laboratories of the University Hospital of Bari.

### Non-invasive hepatic fibrosis scores

Mathematical formulas used for calculating non-invasive liver fibrosis scores are summarised in Supplementary Table S1. APRI score has been developed as a non-invasive index for the assessment of liver fibrosis in patients with viral hepatitis and represents an alternative to liver biopsy in the follow-up of patients with hepatitis C and liver cirrhosis in an outpatient setting^36^. The adopted cut-offs are APRI < 0.5 to identify a fibrosis—free liver, APRI ≥ 0.5 for liver fibrosis and APRI ≥ 1.5 for probable cirrhosis^36^. FIB-4 is a non-invasive score used to assess liver fibrosis in outpatient patients with Hepatitis-C Virus, NAFLD, and other liver complications^37^, although a high false positive rate has been detected for advanced fibrosis in older patients^38^. The validated cut-offs are as followed: FIB-4 < 1.45 for no or moderate fibrosis 1.45 < FIB-4 < 3.25 for moderate fibrosis, FIB-4 ≥ 3.25 for extensive fibrosis or cirrhosis^37^. mFIB-4 index was elaborated on as an instrument to assess the stage of liver fibrosis in patients with chronic hepatitis B or C. However, it is used for the detection of advanced liver fibrosis in all patients with chronic liver disease^39^. AARPRI derived from FIB-4 by removing age and can be considered as one of the most reliable scores in the diagnosis of advanced stages of liver fibrosis, thus also being proposed as a predictor for chronic liver disease-associated complications^32,40^.

### Statistical analysis

Descriptive statistical analyses of the study sample were performed, and their results were expressed as mean ± standard deviation (SD) for numerical data, in counts and percentages for categorical data. Comparisons of clinical variables between two groups were conducted with Mann–Whitney U Test, while the differences among multiple groups were assessed using the Kruskal Wallis test followed by post-hoc analysis (Fisher test). All reported p-values (p) were based on two-sided tests and compared to a significance level of 5%.

Cut-off point analysis was used to determine the optimal value of variables differentiating patients who developed cancer from women who did not. In particular, the crucial point was defined by the largest distance from the diagonal line of the ROC curve. Empirical ROC curves were plotted along with calculation of the AUC with 95% confidence intervals and one-sided upper p-values for null hypothesis AUC = 0.5. Youden’s Index, or equivalently, the highest Sensitivity + Specificity, was used to determine the optimal cut-off of each score for cancer prediction. The variable cut-offs were then analysed for sensitivity, specificity, accuracy, negative predictive value (NPV) and positive predictive value (PPV). p < 0.05 were considered significant. Contingency table and chi-square tests (Fisher’s exact tests when required) were used to study the association between categorical variables and calculating OR with their relative 95% confidence intervals (CI).

All analyses were performed using the NCSS 12 Statistical Software, version 12.0.2018 (NCSS, LLC Company, Kaysville, UT, USA) and GraphPad Prism, version 9.1.0 (GraphPad Software; San Diego, CA, USA).

### Ethics approval statement

The study was approved by the Ethics Committee (Interdisciplinary Department of Medicine; n.311, MSC/PBMC/2015) of the Azienda Ospedaliero-Universitaria Policlinico di Bari (Bari, Italy) in accordance with the requirements of the Declaration of Helsinki.

### Patient consent statement

Written informed consent for the use of clinical data was obtained from all participants in the study.

### Supplementary Information

Supplementary Table S1.

## Data Availability

The datasets used and/or analysed during the current study are available from the corresponding author on reasonable request.
