# Supplementary material for: AST/ALT-to-platelet ratio (AARPRI) predicts gynaecological cancers: a 8-years follow-up study in 653 women
Source: Sci Rep. 2023 Oct 18;13:17793. doi: 10.1038/s41598-023-44243-y (PMC10584967; doi:10.1038/s41598-023-44243-y)
Supplement: Supplementary file 1 — Supplementary Table S1. [file 41598_2023_44243_MOESM1_ESM.docx]

**Supplementary table S1. Non-invasive scores for liver fibrosis with their associated formulas**

| **Score** | **Formula** |
| --- | --- |
| AARPRI | $\frac{\mathrm{AST}/\mathrm{ALT}\times150}{Platelet Count}$ |
| APRI | $\frac{\mathrm{AST}/{ULN} \times100}{Platelet Count}$ |
| FIB-4 | $\frac{Age\times AST}{Platelet Count \times\sqrt{\mathrm{ALT}}}$ |
| mFIB-4 | $\frac{10 \times Age\times AST}{Platelet Count \times ALT}$ |

Abbreviations: AARPRI: (AST to ALT ratio) to Platelet Ratio Index; APRI: AST-Platelet Ratio Index; FIB-4: Fibrosis-4 index; mFIB-4: modified FIB-4; AST: Aspartate transaminases; ALT: Alanine transaminases; ULN: upper limit of normal values.
